# Prenatal diagnosis of hypospadias with 2-dimensional and 3-dimensional ultrasonography

**DOI:** 10.1038/s41598-019-45221-z

**Published:** 2019-06-17

**Authors:** Xiaohua Li, Aqing Liu, Zhonglu Zhang, Xia An, Shaochun Wang

**Affiliations:** Department Of Ultrasonography, Affiliated Hospital of Jining Medical University, Jining, 272000 China

**Keywords:** Urethra, Hypospadias

## Abstract

To compare the prenatal diagnostic performance as well as appearance of ultrasonic details between 2-dimensional ultrasonography (2DUS) combined with 3-dimensional ultrasonography (3DUS) and 2DUS alone for hypospadias. A total of 47 fetuses were enrolled and examined by 2DUS and then 3DUS. Postnatal follow-up data were obtained and 28 cases were confirmed of hypospadias. Although not statistically significant, there was a trend toward higher AUC (0.85 vs. 0.76; p = 0.08), ACC (85.1 vs. 76.6%; p = 0.22), SEN (85.7 vs. 78.6%; p = 0.63), and SPE (84.2 vs. 73.7%; p = 0.50) for 2DUS combined with 3DUS compared with 2DUS alone. The agreement between both methods was moderate [kappa = 0.592]. Both modalities showed accurately the short penis and blunt tip of the penis. 2DUS in combination with 3DUS showed more cases in other detailed features, such as “chordee”, a “hooded” incomplete prepuce, and so on. Overall 2DUS combined with 3DUS showed a trend toward higher performance compared with 2DUS alone for the diagnosis of hypospadias, although the difference was not statistically significant. 3DUS is a useful complement for 2DUS in the diagnosis of fetal hypospadias and may provide more detailed information related to its diagnosis and prognosis.

## Introduction

Hypospadias refers to urinary tract ectopic opening at any position along penile ventral to the perineum, the most common deformity for male genitalia, the incidence for live births is 0.2–4.1/1000^1^. For newborns, the diagnosis of hypospadias is self-evident, while prenatal diagnosis relies mainly on ultrasonography. With the development of ultrasonic technology and improvement of detector’s resolution, traditional 2-dimensional ultrasonography (2DUS) has evaluated fetal genitals better than before, but it still has some shortcomings, such as most of 2D criterias for fetal hypospadias are indirect signs, and ultrasonic image is greatly influenced by the operator’s experience and fetal position, so prenatal misdiagnosis is common. Literatures have reported that 3-dimensional ultrasonography (3DUS) may show clearly the shape of the penis and the relationship between the penis and scrotum, contributing to prenatal diagnosis of fetal hypospadias^2–5^. However, most of these were described in case reports, and as far as we know, no study has compared the diagnostic performance of fetal hypospadias between 2DUS and 3DUS. In this report, receiver operating characteristic (ROC) curve analysis was performed, and we compare the area under the ROC curve (AUC), diagnostic accuracy (ACC), sensitivity (SEN), specificity (SPE), and detailed ultrasonic findings related to the diagnosis of hypospadias between 2DUS in combination with 3DUS and 2DUS alone.

## Materials and Methods

### Selected pregnant women and their basic information

From July 2012 to October 2018, 34 fetuses suspected of having hypospadias on the basis of ultrasonographic findings and 13 with a family history were included in this study, of which 25 were from our hospital and 22 were from other hospitals. All the pregnant women were referred to our tertiary center for US examination, 41 were singleton pregnancies, while 6 were twin pregnancies. The pregnant women were aged between 20 and 43, the median age 31, and the gestational age was between 21 weeks and 35 weeks, the median gestational age 29 weeks 5 days. The gestational age was determined by the last menstruation date or ultrasonography at an early gestational stage.

Among the 47 pregnant women, 39 had conceived naturally, 4 by ovulation induction treatment with clomiphene citrate, and 4 through vitro fertilization (IVF).

### 2DUS and 3DUS evaluation

Three fellowship trained sonographers who had a minimum of 15 years of experience in ultrasonic scanning and diagnosing fetal urinary anomalies participated in the study. In order to eliminate bias, the chances that three researchers perform 2DUS or 2DUS in combination with 3DUS were random.

Transabdominal 2DUS was performed by one researcher using a Voluson E8 scanner equipped with a 3- to 5-MHz convex probe(GE Healthcare, Milwaukee, WI). Another researcher, masked to the initial 2DUS findings, used a SAMSUNG UGEO WS80A (Samsung electronics, Korea) or Voluson E8 (GE Healthcare, Milwaukee, WI) scanner with a 4- to 7-MHz abdominal volume probe and a 3- to 5-MHz convex probe to perform combined 2DUS and 3DUS along with an analysis of the 3DUS volume. Both sets of examiners were blinded to the other’s results. An obstetric setting with safe mechanical and thermal indices was used for acquiring the 2DUS images and 3DUS volumes, which were stored on a hard disk for subsequent analysis. Pregnant women were in dorsal position or lateral position usually. If the perineum and genitalia were obscured, the area was reassessed later during the examination.

Investigators were asked to make diagnosis of hypospadias (“yes”, “no”, or “unable to determine”) and indicate the presence of specific findings that have been reported to be associated with its diagnosis. These findings included the following: (1) a blunter bulbous tip of penile shaft; (2) a short penis; (3) various degrees of abnormal curvature of the penis (chordee); (4) dorsal prepuce is thickened obviously, and in lack of ventral prepuce; (5) ventral deflection of the urinary stream; (6) penoscrotal transposition; (7) a groove on ventral penis.

### Statistical analysis

Categorical variables are shown as frequencies and percentages. To compare the diagnostic performance of 2DUS combined with 3DUS and 2DUS alone for hypospadias, receiver operating characteristic (ROC) curve analysis was performed, and the area under the ROC curve (AUC) with 95% confidence interval (CI) was calculated and compared between the two modalities. Accuracy (ACC), sensitivity (SEN), specificity (SPE), Youden Index (YI), positive predictive value (PPV), and negative predictive value (NPV) as well as 95% CI of each estimate were calculated. Then SEN, SPE, and ACC were compared between the two modalities using McNemar’s test.

The Cohen’s kappa test was used to assess the agreement between the two modalities in the diagnosis of hypospadias. Kappa value ≥ 0.75 means perfect agreement, 0.75 > Kappa value ≥ 0.4 means moderate agreement, Kappa value <0.4 means poor agreement.

Data were analyzed using the statistical packages R (The R Foundation; http://www.r-project.org; version 3.4.3) and Empower (R) (www.empowerstates.com, X&Y solutions, inc. Boston, Massachusetts). Power calculation was performed using Pass 15.0.5 software (NCSS, Kaysville, Utah, USA). The level of significance was set at P < 0.05, and all the tests were two-tailed.

### Ethics approval and consent to participate

The study protocol was approved by Jining Medical University Affiliated Hospital’s Ethics Committee. Written informed consent was obtained from each patient after a careful explanation of the purposes of the study.

## Results

### Postpartum follow-up findings

Postnatal follow-up data were obtained in all 47 fetuses. 42 were live born, with full-term normal delivery in 30 cases, cesarean delivery in 8 cases, and premature birth in 4 cases, and 5 were terminated. Physical examinations were performed by experienced pediatric surgeons on 42 newborns, and autopsies were performed on all 5 terminated fetuses to determine the presence or absence of hypospadias. Full chromosomal analysis was performed in 30 fetuses, revealing karyotypes of 46, XY (27 cases), 47, xxy (2 case), and small Y (1 case). The karyotypes of the other 17 fetuses were not available.

Of 47 fetuses, 28 were confirmed with hypospadias after birth or induced labor (anterior, 9 cases; middle, 7 cases; and posterior, 12 cases). Of other 19 fetuses without hypospadias, 8 had normal male genitalia, 2 had normal female genitalia with large labia, 4 had pure penoscrotal transposition, 4 had short penis, and 1 had 46,XX DSD (disorder of sex development).

Other associated postnatal anomalies in the 28 fetuses with hypospadias included penoscrotal transposition (19 cases), undescended testis (12 cases), unilateral inguinal hernia (4 cases), short limbs (1 case), facial deformity (1 case), and ventricular septal defect (2 cases).

### Diagnostic agreement between 2DUS in combination with 3DUS and 2DUS alone

Among the 28 fetuses confirmed as having hypospadias, 22 had a correct diagnosis by 2DUS, whereas 24 had a correct diagnosis by the combination of 2DUS and 3DUS. With 2DUS alone, 14 of 19 fetuses without hypospadias had a correct diagnosis, and with the combination of 2DUS and 3DUS, 16 had a correct diagnosis (Table 1). 35 fetuses had a correct diagnosis by the two methods, 6 fetuses had a wrong diagnosis by both methods, and the results of the two methods were inconsistent in 6 cases. The agreement for the diagnosis of hypospadias between the two methods was moderate [kappa = 0.592; 95% confidence interval (CI): 0.284–0.845]. Interestingly, 3 cases missed by both methods were confirmed as having anterior hypospadias after birth.

**Table 1:** Summary of prenatal diagnosis with 2-dimensional ultrasonography alone and in combination with 3-dimensional ultrasonography.

Modality	hypospadias		no hypospadias	
	Correct	Incorrect	Correct	Incorrect
2DUS	22	6	14	5
2DUS + 3DUS	24	4	16	3

3DUS indicates 3-dimensional ultrasonography; and 2DUS, 2-dimensional ultrasonography.

### Diagnostic performance of 2DUS in combination with 3DUS and 2DUS alone

**Figure 1:**
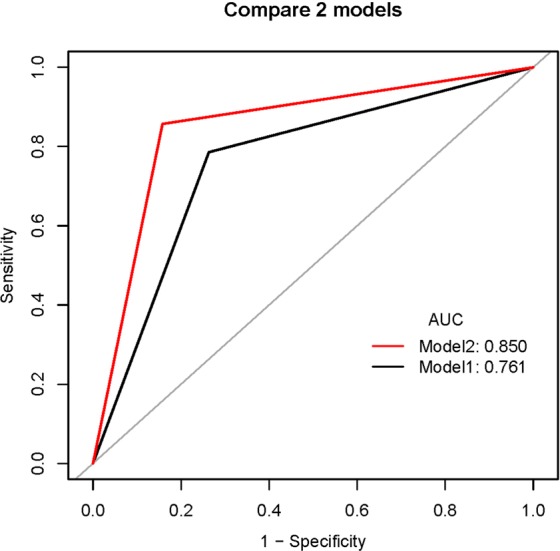
Comparison of the area under receiver operating characteristics curves (ROC) for 2DUS combined with 3DUS and 2DUS alone. There was a trend toward higher AUC (0.85 vs. 0.76; p = 0.08) for 2DUS combined with 3DUS compared with 2DUS alone, although the difference was not statistically significant. Model 1 indicates 2DUS alone, and Model 2, 2DUS combined with 3DUS.

The AUC, ACC, SEN, SPE, YI, PPV, and NPV of 2DUS in combination with 3DUS and 2DUS alone to diagnose fetal hypospadias are reported in Table 2. The AUC values of 2DUS in combination with 3DUS and 2DUS alone were 0.85 (95% CI, 0.74–0.96) and 0.76 (95% CI, 0.63–0.89), respectively, with no significant difference between the two modalities (p = 0.08) (Fig. 1). Although not statistically significant, there was a trend toward higher AUC (0.85 vs. 0.76%; p = 0.08), ACC (85.1 vs. 76.6%; p = 0.22), SEN (85.7 vs. 78.6%; p = 0.63), and SPE (84.2 vs. 73.7%; p = 0.50) for 2DUS combined with 3DUS compared with 2DUS alone.

**Table 2:** Comparison of diagnostic performances for hypospadias between 2DUS alone and in combination with 3DUS.

Parameter	2DUS	2DUS + 3DUS	P-value
AUC (95% CI)	0.76 (0.63–0.89)	0.85 (0.74–0.96)	0.08
SEN (%) (95% CI)	78.6 (59.1–91.7	85.7 (67.3–96.0)	0.63
SPE (%) (95% CI)	73.7 (48.8–90.9)	84.2 (60.4–96.6)	0.50
ACC (%) (95% CI)	76.6 (62.0–87.7)	85.1 (71.7–93.8)	0.22
Y I (95% CI)	0.52 (0.08–0.83)	0.70 (0.28–0.93)	
PPV (95% CI)	0.81 (0.62–0.94)	0.89 (0.71–0.98)	
NPV (95% CI)	0.70 (0.46–0.88)	0.80 (0.56–0.94)	

3DUS indicates 3-dimensional ultrasonography; 2DUS, 2-dimensional ultrasonography; ACC, accuracy; SEN, sensitivity; SPE, specificity; YI, Youden Index; PPV, positive predictive value; and NPV, negative predictive value.

This study was not initially powered on a noninferiority hypothesis because the prenatal diagnosis of hypospadias by 2DUS and 3DUS is rare. So a post hoc power calculation was performed for the primary endpoint of the difference in diagnostic ACC of the two modalities, and a sample size of 47 pairs achieved 19.6% power to detect an odds ratio of 0.200 (the actual ratio in this study) using a two-sided McNemar test with a significance level of 0.050.

### Detailed findings of 2DUS in combination with 3DUS and 2DUS alone

21 fetuses had a correct diagnosis of hypospadias both by 2DUS alone and 2DUS in combination with 3DUS. Detailed findings of both methods in these fetuses were compared with neonatal examination results or autopsy results. Both 2DUS and 2DUS in combination with 3DUS showed accurately and clearly the short penis and the blunt tip of the penis in the fetus with hypospadias. 2DUS in combination with 3DUS showed more cases in other detailed features than 2DUS, such as chordee (ventral incurvation of the penis), the thickened dorsal prepuce, penoscrotal transposition, and the appearance of urethral groove. But 3DUS had no advantage in the observation of anomalous urinary stream during fetal micturition. The prenatal detection rate of urethral groove was relatively low for both methods (Table 3).

**Table 3:** Comparison of detailed specific findings of 2DUS combined with 3DUS and 2DUS alone with the results of inspection of the newborn or autopsy.

The detailed specific findings	2DUS, n (%)	3DUS + 2DUS, n (%)	The results of inspection of the newborn or autopsy, (n)
A blunt tip at the penile shaft	21 (100)	21 (100)	21
A short penile shaft	17 (100)	17 (100)	17
Ventral incurvation of the penis	12 (70.6)	15 (88.2)	17
Thickened dorsal prepuce	14 (77.8)	16 (88.9)	18
Penoscrotal transposition	13 (76.5)	15 (88.2)	17
The appearance of urethral groove	9 (56.3)	11 (68.8)	16
The anomalous urinary stream	11 (52.3)	0 (0)	21

2DUS indicates 2-dimensional ultrasonography; and 3DUS, 3-dimensional ultrasonography.

### Comparation of 3DUS image with postnatal photo

**Figure 2:**
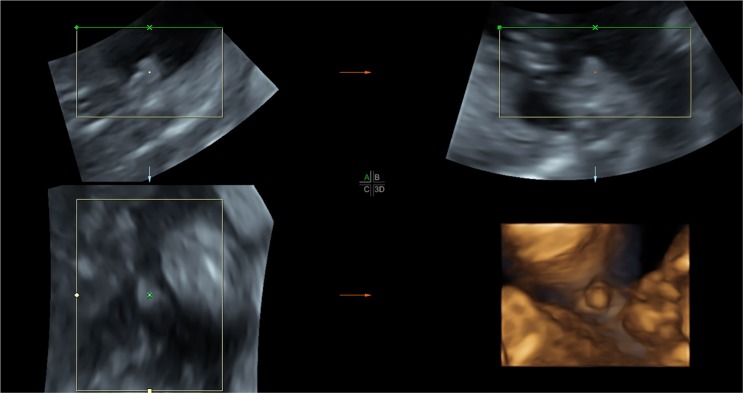
Three-dimensional multiplanar images of the external genitalia at 21 weeks of gestation in longitudinal (top left), axial (top right), and coronal (bottom left) views allow construction of a surface-rendered image (bottom right). A short and incurved penile shaft with a blunt tip was shown in three orthogonal planes simultaneously. Three-dimensional image in surface-rendered view shows excess dorsal prepuce (dorsal hood).

**Figure 3:**
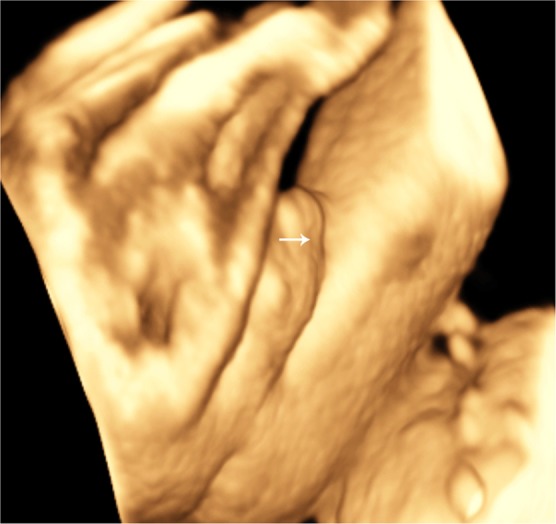
The ventral view of the penis was demonstrated by surface-rendered three-dimensional image at 28 weeks of gestation. The short penis with a shallow groove in the direction of up and down resulted from the incomplete fusion of the urogenital folds is shown (arrow).

**Figure 4:**
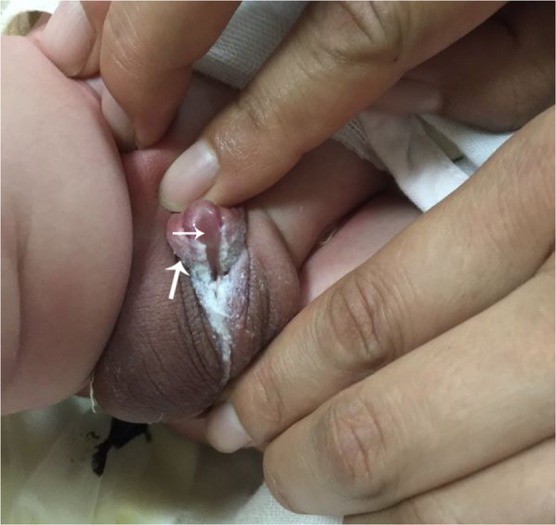
Photograph of the newborn confirms the hypospadias. The dorsal thickened prepuce (big arrow) and ventral urethral groove (small arrow) of the penis are shown.

The 3D images of multiplanar and surface-rendered mode are shown in Figs 2 and 3 respectively. Figure 4 shows photograph of the newborn with hypospadias.

## Discussion

Urologic classification of hypospadias is based on meatal position: anterior (glandular or coronal), occurring in approximately 50%; middle (penile shaft), constituting 30%; and posterior, accounting for the remaining 20%. A spectrum of abnormalities, including chordee, a “hooded” incomplete prepuce, and an abortive corpora spongiosum, are commonly associated with hypospadias. The exact etiology of this disorder remains largely unknown. Literatures have reported that the origin of hypospadias seems to be multifactorial, and several risk factors have been identified, such as genetic predisposition, placental insufficiency, and substances that interfere with natural hormones^6^.

Conventional sonographic criteria for diagnosis of hypospadia are a blunt tip at the penile shaft rather than a normal pointed morphology, a short penile shaft, abnormal curvature of the penis, and an anomalous urinary stream^1,7^. Meizner^7^ *et al*. described the“ tulip sign” as an US clue for the in utero diagnosis of severe hypospadias and the tulip was formed by a ventrally orientated penis located between the scrotal fold. Other studies have suggested that 3DUS may provide a systemic evaluation of the external genitalia and contribute to prenatal diagnosis of hypospadias by providing more detailed and realistic images^2–5^. However, most of these were described in case reports, and to our knowledge no studies have compared the diagnostic efficiency between 2DUS and 3DUS. In this study, we compared the diagnostic efficiency of 2DUS in combination with 3DUS and 2DUS alone in the diagnosis of hypospadias, and the results showed that there was a trend toward higher AUC, ACC, SEN, and SPE for 2DUS combined with 3DUS compared with 2DUS alone, although the difference was not statistically significant (all P > 0.05). The agreement for the diagnosis of hypospadias between the two methods was moderate [kappa = 0.592]. And we believe that when the results of the two methods are inconsistent in practical work, the results of 2DUS combined with 3DUS may be more reliable.

Advantages of 3DUS are multiplanar and surface-rendering mode. The multiplanar view helps us make a better correlation of the anomaly in the 3 orthogonal planes simultaneously, providing more details. And surface mode can provide a “sculpture-like” picture that can be rotated in all directions, permitting inspection from different angles. In this study, 2DUS combined with 3DUS showed more detailed features than 2DUS alone, such as the blunt tip at the penile shaft, the ventral incurvation of the penis, the “hooded” incomplete prepuce, penoscrotal transposition, and the appearance of urethral groove. Thus 3DUS may show more detailed information and improve the confidence of diagnosis for hypospadias in some cases, when it is difficult to clarify a diagnosis by 2DUS alone. And Moreover, the realistic images provided by 3DUS can be shared with pediatric surgeons and parents, who may know more about surgery and prognosis of this abnormality, helping them make the best decision. Therefore, 3DUS is a useful complement for 2DUS in the diagnosis of fetal hypospadias.

Although 3DUS is helpful in the diagnosis of hypospadias, it remains difficult to make a correct diagnosis in certain cases. In our study, 3 cases were misdiagnosed as hypospadias prenatally by 2DUS alone and 2DUS in combination with 3DUS. One case was confirmed with penoscrotal transposition combined with congenital testicular dysgenesis syndrome after birth, with short penis like the clitoris and normal urethral meatus. One case was confirmed with penoscrotal transposition with a short penis. The 2DUS and 3DUS features of genitals were similar to”tulip sign” in the above two cases, and it was difficult to correctly diagnose the deformity. The other one was confirmed with female pseudohermaphroditism after birth. Although sonographic fetal sex determination is feasible in most pregnancies, in other cases, it may pose difficulties. And it is difficult to identify a micropenis from an enlarged clitoris and a split scrotum from an enlarged labia, and the descended testes and the distance between the posterior wall of the bladder and anterior wall of the rectum may be useful in some cases^8,9^. Moreover amniocentesis is an invasive method to check fetal chromosomes and determine the sex of the fetus. In our study, anterior hypospadias had the highest proportion of missed cases compared with other types, possibly due to its atypical ultrasonic signs. Of the 6 cases missed by 2DUS alone, 5 were confirmed with anterior hypospadias after birth or induced labor. And all the 4 cases missed by 2DUS in combination with 3DUS were diagnosed as anterior hypospadias. Fortunately, anterior hypospadias has a relatively good prognosis, especially when the penis length is normal.

We recognize the limitation of the current study. First, 2DUS was used to identify possible cases of hypospadias, that were then assessed by 2DUS combined with 3DUS. This design is inherently biased against 3DUS as there may (or may not) have been a higher pick-up rate had 3D ultrasound been used in an unselected population. Another limitation was the relatively small number of cases, and the power was really quite low, thus limiting the statistical strength of the study. And a larger sample size is needed to verify the results of this study in the future.

## Conclusion

In conclusion, 2DUS combined with 3DUS showed a trend toward higher performance compared with 2DUS alone for the diagnosis of hypospadias, although the difference was not statistically significant. 3DUS is a useful complement for 2DUS in the diagnosis of fetal hypospadias and may provide more detailed information related to its diagnosis and prognosis. A multicenter prospective study with sufficient sample size is needed to validate our findings, and confirm the effectiveness of 3DUS and its ability to improve the prenatal diagnosis of hypospadias compared with 2DUS prior to its appropriate application in clinical practice.

## Supplementary information

Supplementary Information

## Data Availability

All data generated or analysed during this study are included in this published article and its Supplementary Information files.
