# Supplementary material for: Prenatal diagnosis of hypospadias with 2-dimensional and 3-dimensional ultrasonography
Source: Sci Rep. 2019 Jun 17;9:8662. doi: 10.1038/s41598-019-45221-z (PMC6572849; doi:10.1038/s41598-019-45221-z)

Prenatal diagnosis of hypospadias with 2-dimensional and 3-dimensional ultrasonography

Xiaohua Li, Aqing Liu, Zhonglu Zhang, Xia An, Shaochun Wang

Table 1. Summary of prenatal diagnosis with 2-dimensional ultrasonography alone and in combination with 3-dimensional ultrasonography

Modality		2DUS+3DUS	
		Correct	Incorrect
2DUS	Correct	35	1
	Incorrect	5	6

Table 2. Descriptions of 21 cases of hypospadias diagnosed correctly both by 2DUS alone and 2DUS in combination with 3DUS

Case	GA, wk	Prenatal 2DUS and 3DUS Findings	Associated Postnatal Anomalies	Karyotype
1	27	A blunt tip at the penile shaft, the ventral incurvation of the penis, thickened dorsal prepuce, the appearance of urethral groove, the anomalous urinary stream during fetal micturition	Middle hypospadias, bilateral undescended testis	46XY
2	30	A blunt tip at the penile shaft, a short penile shaft, the ventral incurvation of the penis, thickened dorsal prepuce, penoscrotal transposition	Posterior hypospadias, cleft lip	46XY (Small Y)
3	33	A blunt tip at the penile shaft, a short penile shaft, thickened dorsal prepuce, the appearance of urethral groove, the anomalous urinary stream during fetal micturition	Posterior hypospadias, ventricular septal defect	46XY
4	31	A blunt tip at the penile shaft, a short penile shaft, the ventral incurvation of the penis, thickened dorsal prepuce, penoscrotal transposition	Posterior hypospadias, left undescended testis	46XY
5	31	A blunt tip at the penile shaft, a short penile shaft, penoscrotal transposition, the appearance of urethral groove, the anomalous urinary stream during fetal micturition	Anterior hypospadias	46XY
6	34	A blunt tip at the penile shaft, a short penile shaft, the ventral incurvation of the penis, thickened dorsal prepuce,	Posterior hypospadias	46XY

		penoscrotal transposition, the appearance of urethral groove		
7	34	A blunt tip at the penile shaft, a short penile shaft, the ventral incurvation of the penis, the appearance of urethral groove, the anomalous urinary stream during fetal micturition	Anterior hypospadias, right undescended testis, left renal deficiency	NA
8	32	A blunt tip at the penile shaft, a short penile shaft, the ventral incurvation of the penis, thickened dorsal prepuce, penoscrotal transposition, the appearance of urethral groove, the anomalous urinary stream during fetal micturition	Posterior hypospadias	46XY
9	29	A blunt tip at the penile shaft, a short penile shaft, the ventral incurvation of the penis, penoscrotal transposition, the appearance of urethral groove	Posterior hypospadias, bilateral undescended testis, right inguinal hernia, ventricular septal defect	46XY
10	26	A blunt tip at the penile shaft， the ventral incurvation of the penis, thickened dorsal prepuce, penoscrotal transposition, the appearance of urethral groove	Anterior hypospadias, bilateral undescended testis,	NA
11	27	A blunt tip at the penile shaft, a short penile shaft, the ventral incurvation of the penis, thickened dorsal prepuce, penoscrotal transposition	Posterior hypospadias, bilateral undescended testis	46XY
12	26	A blunt tip at the penile shaft, a short penile shaft, the ventral incurvation of the penis, penoscrotal transposition, the appearance of urethral groove, the anomalous urinary stream during fetal micturition	Posterior hypospadias, bilateral undescended testis	46XY
13	33	A blunt tip at the penile shaft, thickened dorsal prepuce, penoscrotal transposition	Middle hypospadias	47XXY
14	28	A blunt tip at the penile shaft, a short penile shaft, thickened dorsal prepuce	Middle hypospadias, bilateral undescended testis	46XY
15	27	A blunt tip at the penile shaf, the ventral incurvation of the penis, thickened dorsal prepuce, penoscrotal transposition, the appearance of urethral groove	Middle hypospadias, left inguinal hernia,	46XY
16	22	A blunt tip at the penile shaft, a short	Middle hypospadias,bilateral	47XXY

		penile shaft, the ventral incurvation of the penis, thickened dorsal prepuce, penoscrotal transposition	undescended testis, left strephenopodia, left hydronephrosis	
17	26	A blunt tip at the penile shaft，a short penile shaft, the ventral incurvation of the penis, thickened dorsal prepuce, penoscrotal transposition	Posterior hypospadias, bilateral undescended testis	46XY
18	21	A blunt tip at the penile shaft, a short penile shaft, the ventral incurvation of the penis, thickened dorsal prepuce, penoscrotal transposition, the appearance of urethral groove, the anomalous urinary stream during fetal micturition	Posterior hypospadias	46XY
19	33	A blunt tip at the penile shaft, a short penile shaft, the ventral incurvation of the penis, thickened dorsal prepuce, penoscrotal transposition, the anomalous urinary stream during fetal micturition	Middle hypospadias, right inguinal hernia,	NA
20	29	A blunt tip at the penile shaft, a short penile shaft	Anterior hypospadias, bilateral undescended testis,	46XY
21	22	A blunt tip at the penile shaft, a short penile shaft, thickened dorsal prepuce,tra	Posterior hypospadias	46XY

GA indicates gestational age at diagnosis; and NA, not available.

Table 3. Postpartum follow-up findings

ID	after birth or induced labor	2D	2D+3D
1	1	1	1
2	1	1	1
3	1	1	1
4	1	1	1
5	1	1	1
6	1	1	1
7	1	1	1
8	1	1	1
9	1	1	1
10	1	1	1

11	1	1	1
12	1	1	1
13	1	1	1
14	1	1	1
15	1	1	1
16	1	1	1
17	1	1	1
18	1	1	1
19	1	1	0
20	1	1	1
21	1	1	1
22	1	1	1
23	1	0	1
24	1	0	1
25	1	0	0
26	1	0	0
27	1	0	0
28	1	0	1
29	0	0	0
30	0	0	0
31	0	0	0
32	0	0	0
33	0	0	0
34	0	0	0
35	0	0	0
36	0	0	0
37	0	0	0
38	0	0	0
39	0	0	0
40	0	0	0
41	0	0	0
42	0	0	0
43	0	1	0
44	0	1	0
45	0	1	1
46	0	1	1
47	0	1	1

1 indicates hypospadias, and 0 no hypospadias

Table 4 The AUC of 2DUS combined with 3DUS.

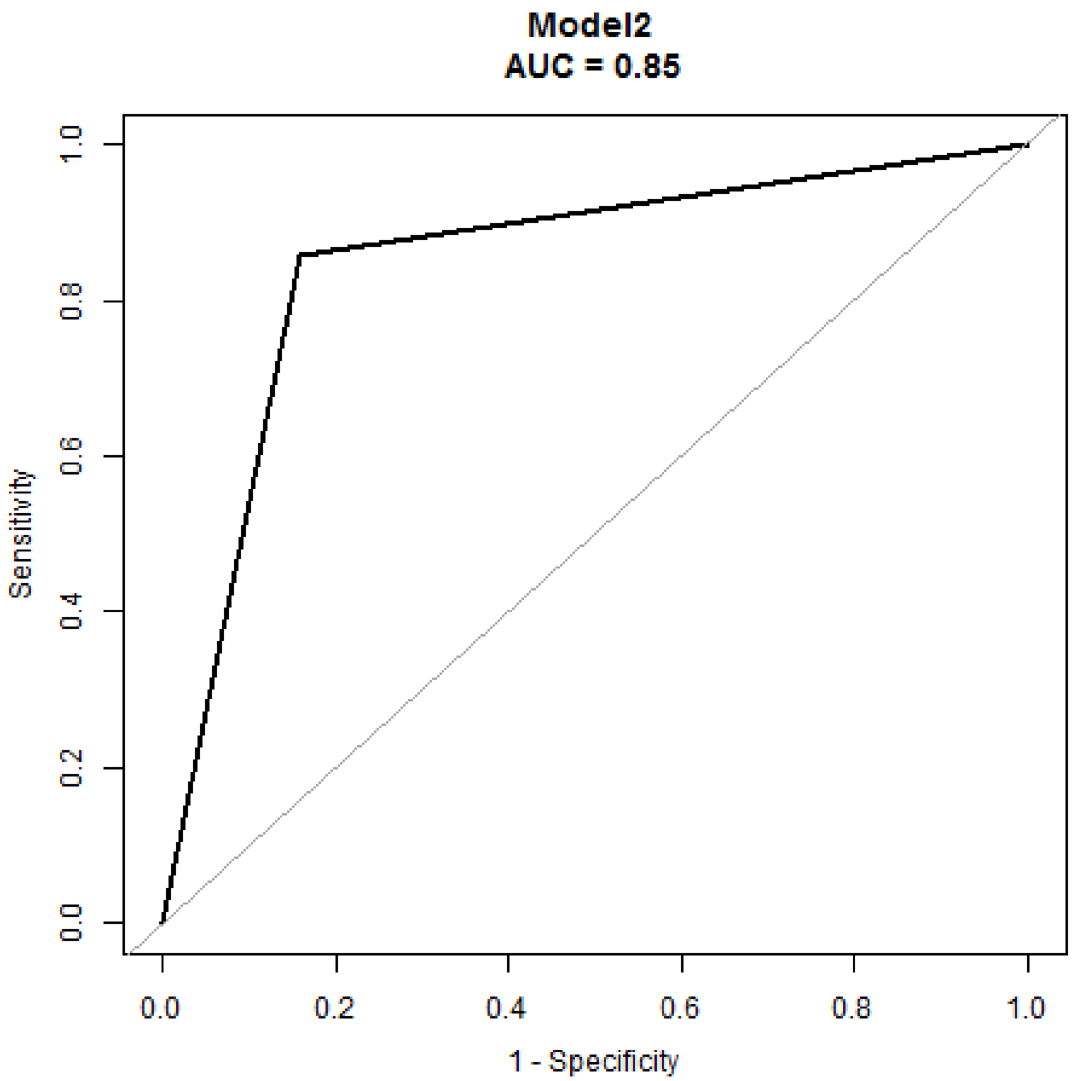

Model 2 indicates 2DUS combined with 3DUS

Table 5. The AUC of 2DUS alone

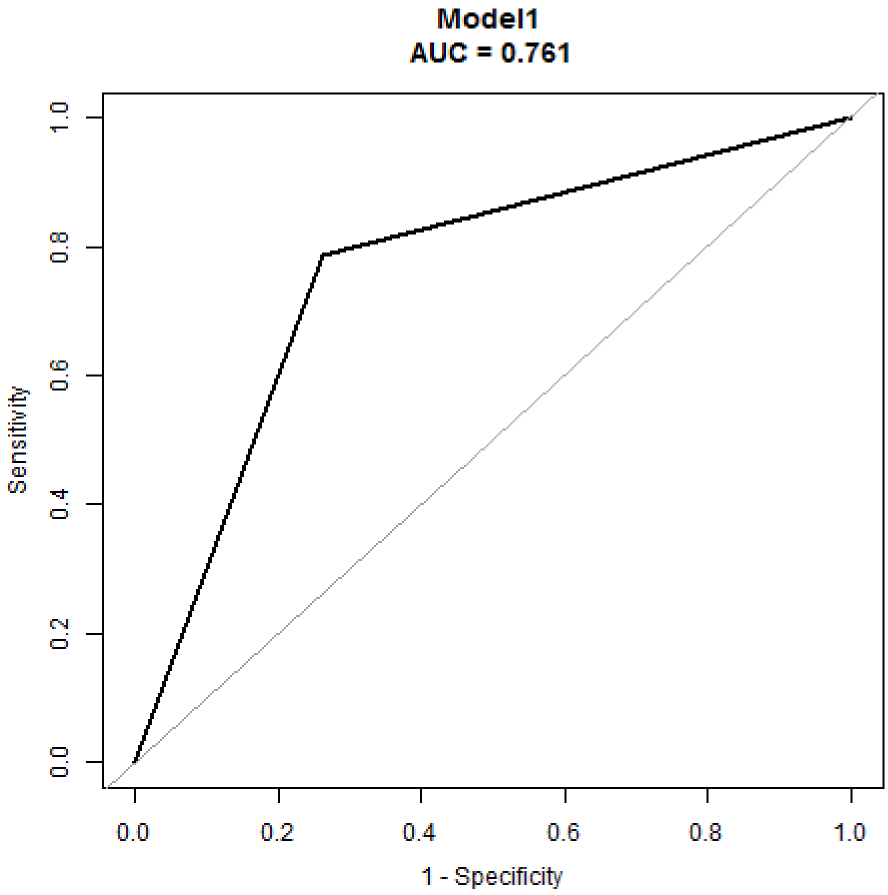

Model 1 indicates 2DUS alone

Table 6. Power on the data (Tests for Two Correlated Proportions (McNemar Test)).

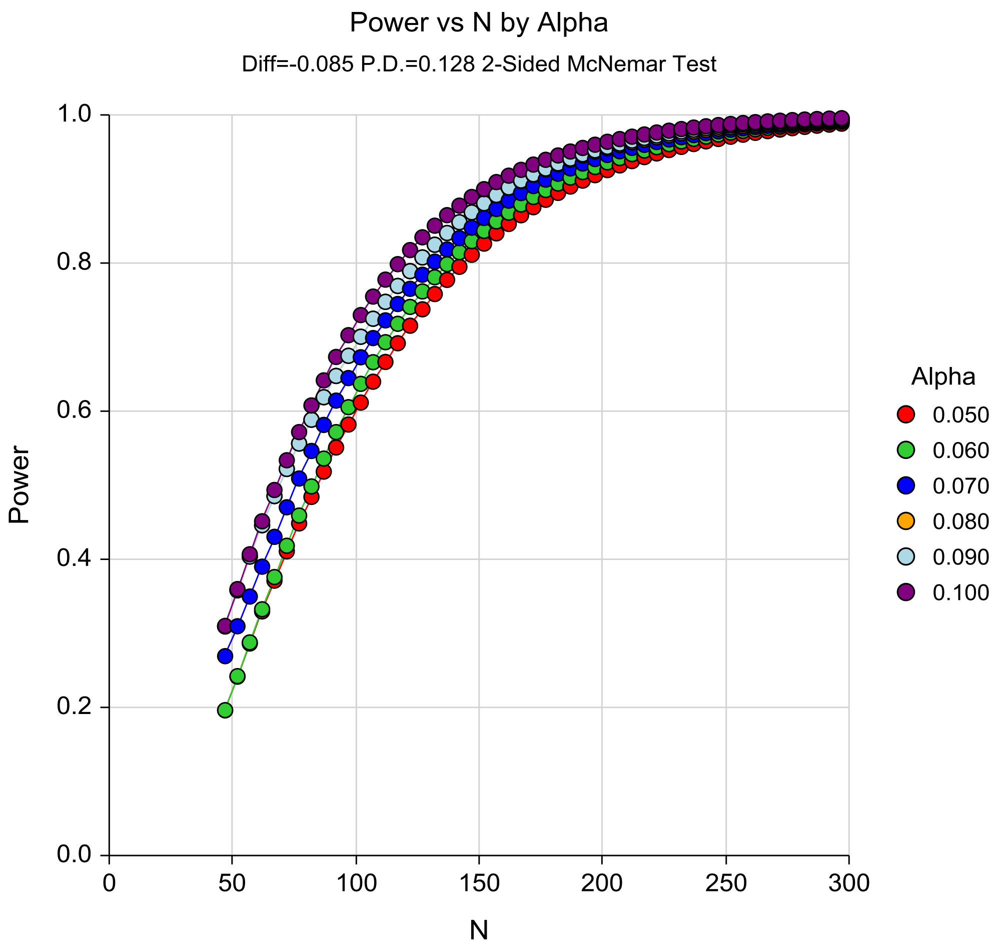

Supplement: Supplementary file 1 — Supplementary Information [file 41598_2019_45221_MOESM1_ESM.pdf]
